# Colorectal Intussusception with an Invasive Adenocarcinoma as Lead Point

**DOI:** 10.5334/jbsr.2536

**Published:** 2021-10-18

**Authors:** Stijn Marcelis, Erik Van Hul

**Affiliations:** 1 AZ Nikolaas, BE

**Keywords:** invagination, intussusception, bowel, colorectal, adenocarcinoma, abdomen

## Abstract

**Teaching Point**: Always look for an underlying (malignant) lead point as cause of a large bowel intussusception in adults.

## Case report

**Figure 1:**
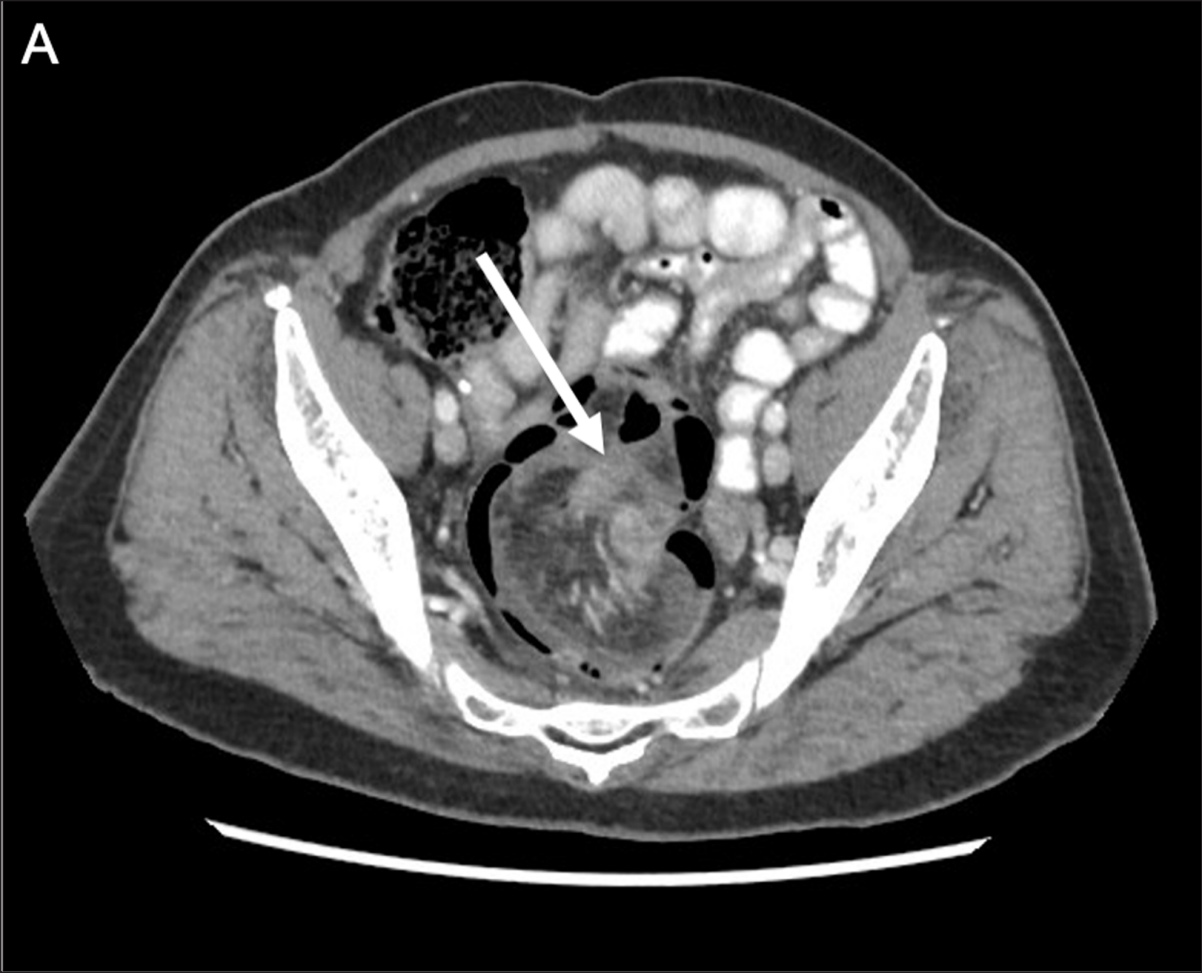


**Figure 2:**
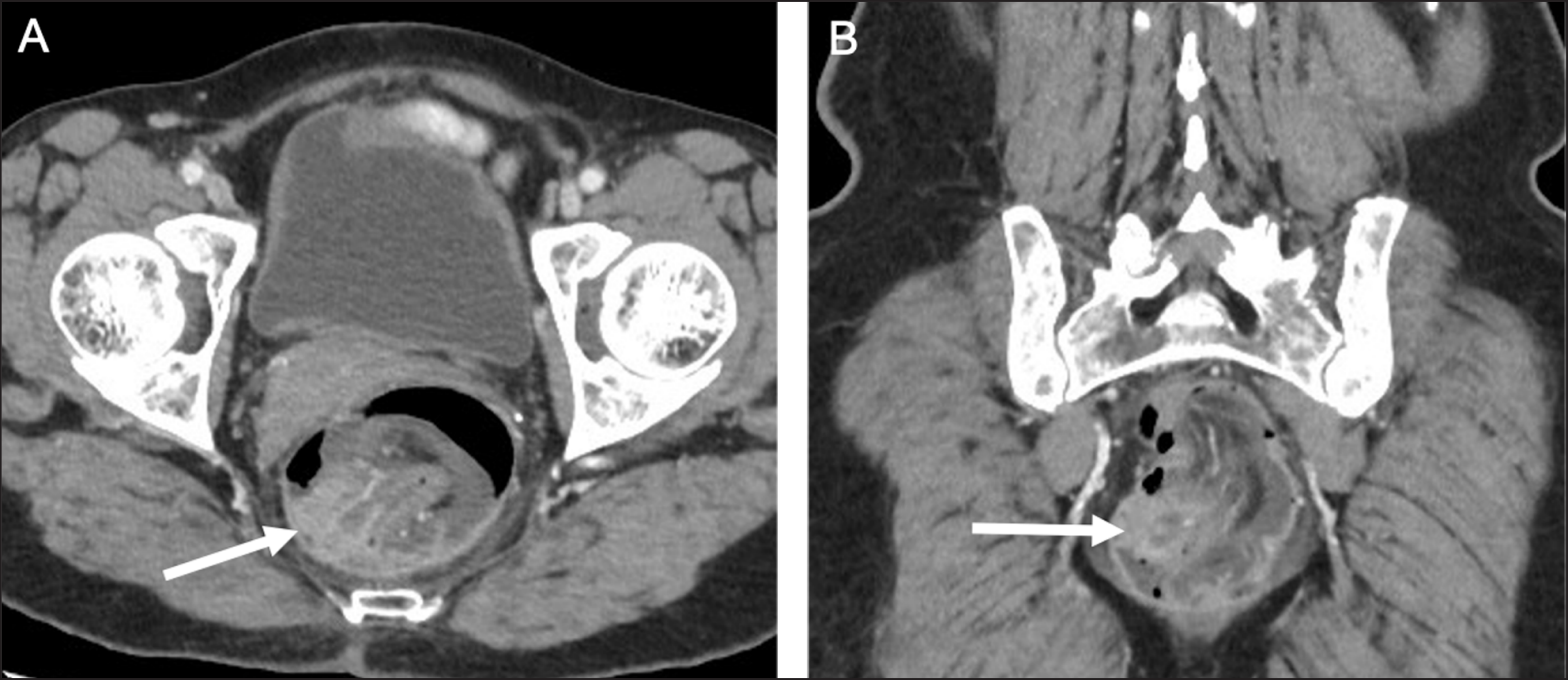


A 78-year-old woman presented at the emergency department with constipation, anal blood loss and impression of anal prolapse. Clinical examination revealed no abnormalities. Contrast-enhanced Computed Tomography (CT) showed a ‘bowel-within-bowel’ configuration with invagination of the adjacent mesentery and vessels at the rectal level (***Figures 1A***, white arrow). The diagnosis of a colorectal intussusception was made. In the distal part of the invaginated segment, there is a soft-tissue mass with enhancement suggestive for a malignancy serving as lead point (***Figure 2A–2B***, white arrow). Resection was performed and histological examination showed an invasive adenocarcinoma.

## Comment

In contrast to children, around 70%–90% of intussusceptions in adults have an underlying cause. Intussusceptions are classified according to location and whether there is a lead point. A lead point is a lesion or variant in the intestine wall that is trapped and dragged into a distal segment to start the intussusception. The lead point can be benign or malignant. Around 90% of intussusceptions occur in small or large bowel with the small bowel being the most common site. Small bowel intussusceptions occur most often without lead point (crohn, celiac sprue, gastric bypass). When a lead point is found, it’s benign in 70% of cases (lipoma, Meckel diverticulum, leiomyoma) and malignant in the remaining cases (often metastasis). Large bowel intussusceptions are associated with malignant lead points in up to 70% of cases. Malignant lesions include primary tumors (adenocarcinoma or lymphoma) and metastasis (melanoma). Benign lesions in large bowel intussusceptions include lipoma, endometriosis and adenomatous polyp [1].

On abdominal CT, a bowel-within-bowel configuration with or without invagination of mesenteric fat and vessels is pathognomonic of intussusception. A mass in the distal part of the invaginated segment serves as an indicator of a lead point. Other findings suggestive for a lead point are long-segment intussusceptions, bowel wall edema and obstruction [1].
